# Supplementary material for: Unraveling enteroendocrine cell lineage dynamics and associated gene regulatory networks during intestinal development
Source: Biol Open. 2025 Oct 21;14(10):bio062083. doi: 10.1242/bio.062083 (PMC12584403; doi:10.1242/bio.062083)
Supplement: Supplementary information [file biolopen-14-062083-s1.pdf]

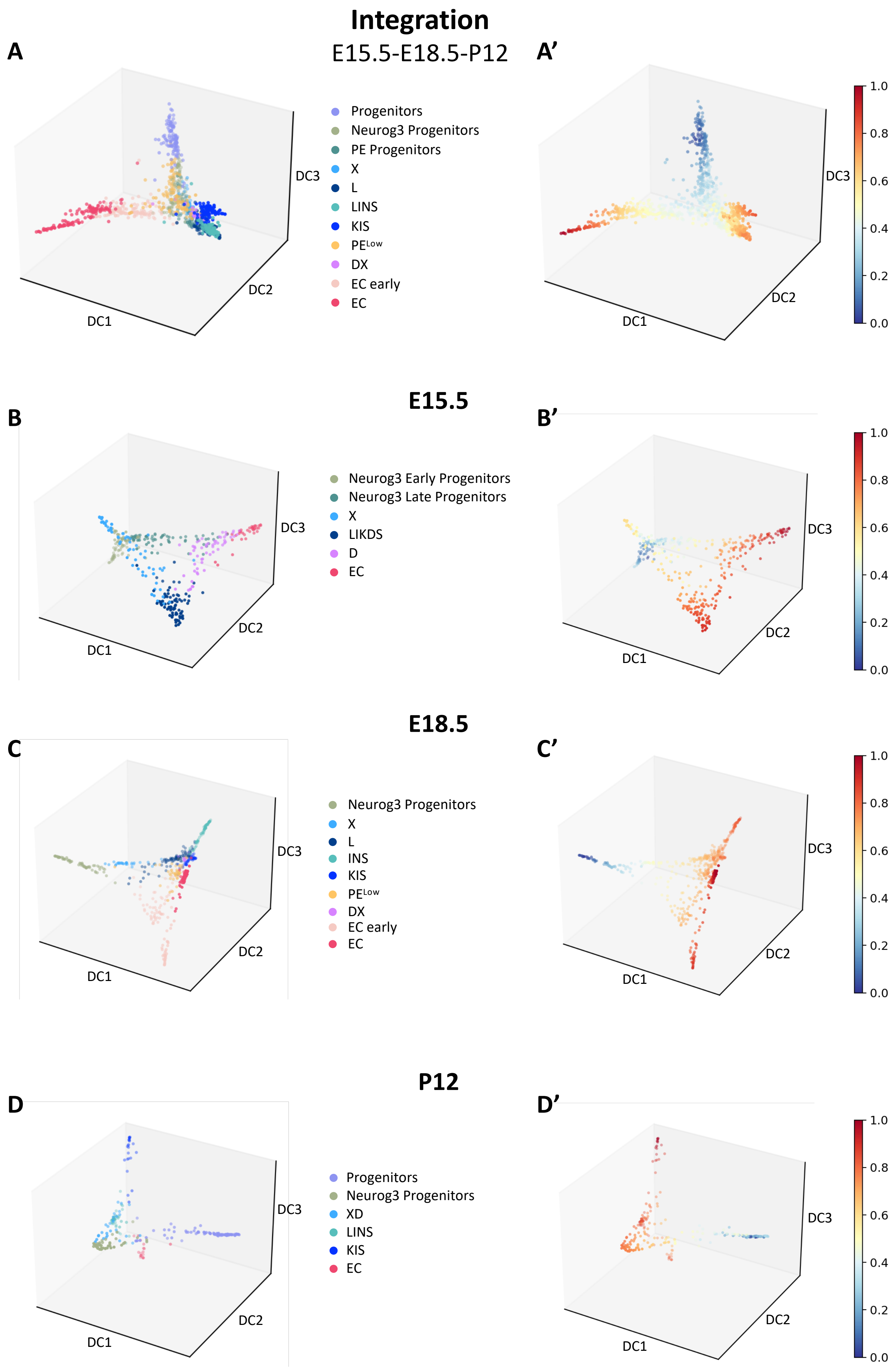

Fig. S1

Biology Open • Supplementary information

Fig. S1. EEC lineage segregation in the mouse small intestine during development. A-D. 3D diffusion map plots illustrating the distribution of mouse small intestinal EECs into different clusters, comprising progenitors and hormone-producing cells, throughout development (embedding of three stages: E15.5, E18.5 and P12) (**A**), or for each stage individually (**B–D**): E15.5, E18.5 and P12, respectively. Clusters are identified by a color code indicated in each panel. **A'-D'.** The pseudotemporal ordering of the cells is shown in the corresponding panels (**A'-D'**). Pseudotime is represented along a gradient from dark blue (early, immature) to red (mature).

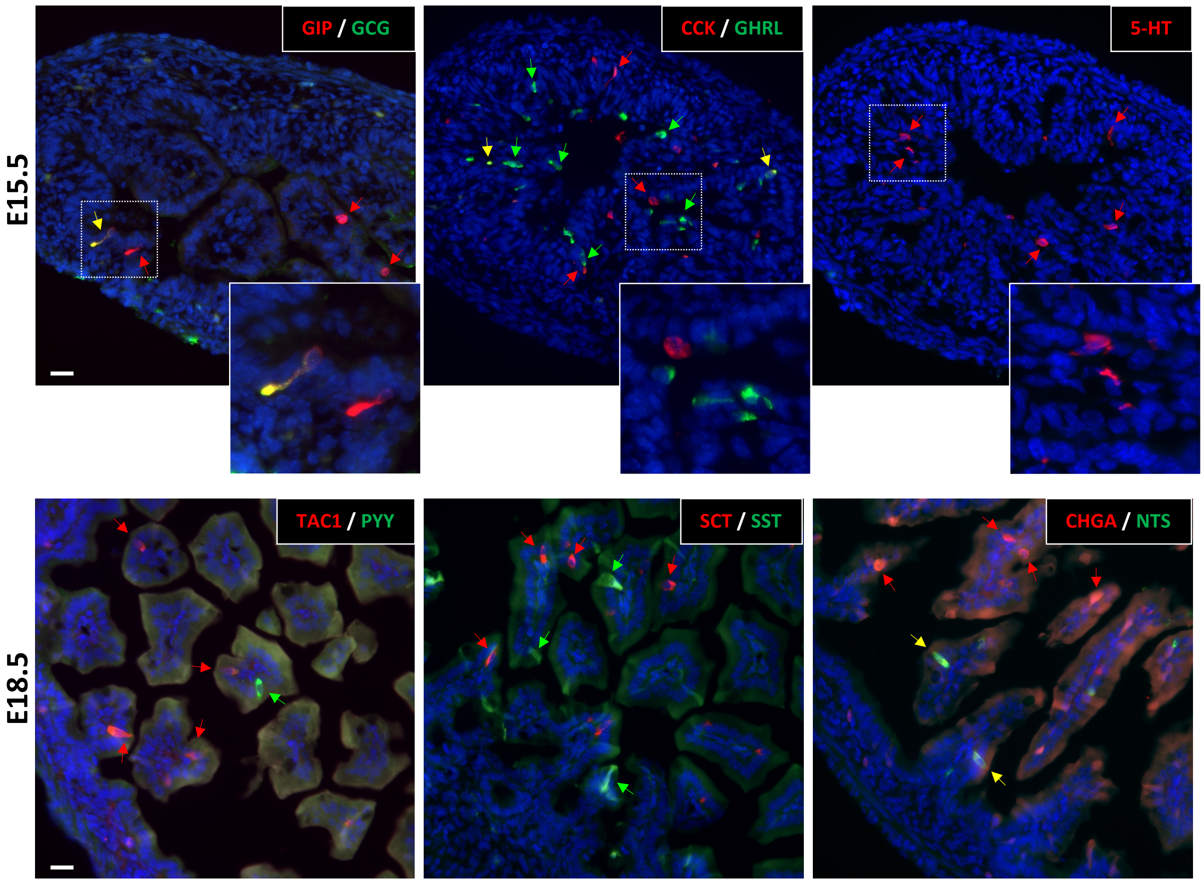

Fig.S2. Expression of hormones in the embryonic mouse small intestine. Immunostaining of GIP/GCG, CCK/GHRL, 5-HT and TAC1/PYY, SCT/SST, CHGA/NTS on E15.5 (upper panels) and E18.5 (lower panels) mouse small intestine cryosections. The markers used are indicated on each panel. Nuclei are stained with DAPI (blue). Red or green arrows point to single-positive cells; yellow arrows point to double-positive cells. Scale bars: 20 μm. Insets in the top panels are shown at 2.5x magnification. (Microscope Leica DM6B-Z).

Biology Open • Supplementary information

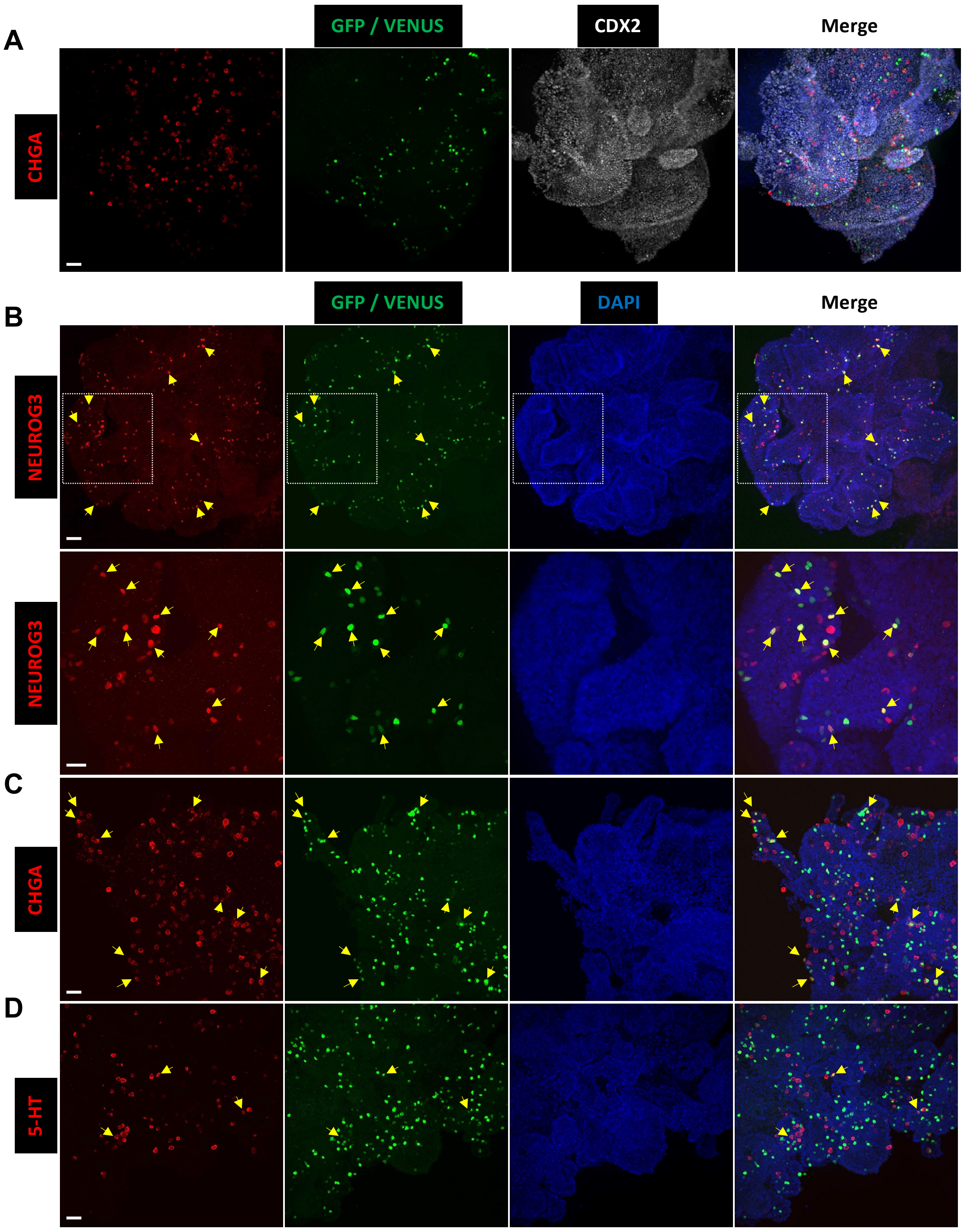

Fig. S3. Progenitor and differentiated EECs in HIOs. Characterization of HIOs (NEUROG3-Venus) by whole-mount immunostainings with progenitor and differentiated EEC markers: CHGA (**A, C**), NEUROG3 (**B**) or 5-HT (**D**) (red); GFP/VENUS (**A-D**) (green); CDX2 (**A**) (white). Nuclei are stained with DAPI (blue). Yellow arrows point to co-positive cells (GFP/VENUS - marker). Insets in (**B**) are shown at higher magnification on the lower panels. The images shown correspond to z-stack maximal intensity projections (Spinning Disk, Leica CSU W1). Scale bars: 100µm.

Biology Open • Supplementary information

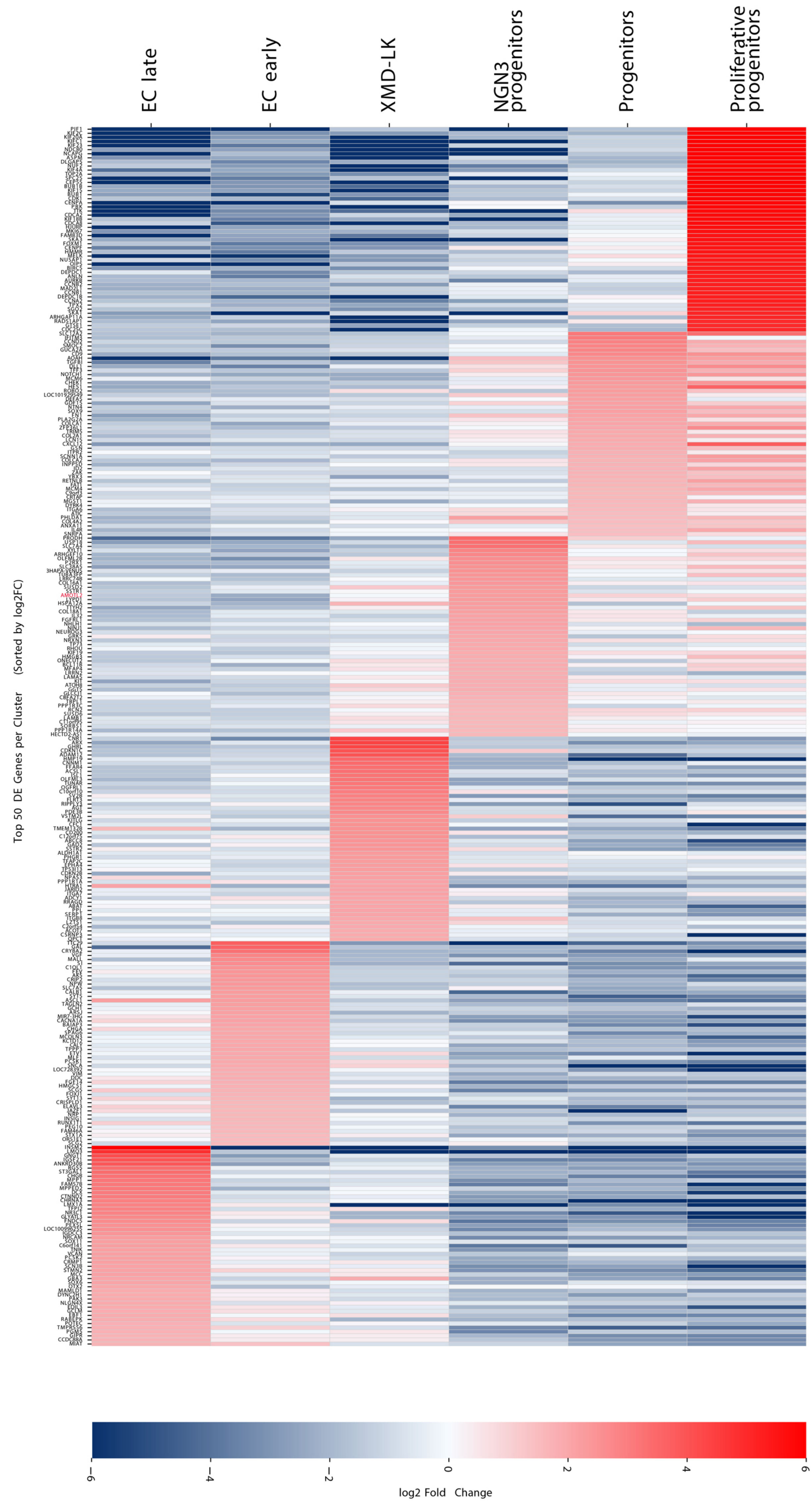

Fig. S4. Top 50 differentially expressed genes in HIOs cell clusters (ranked by Fold Change). Heatmap showing the fold changes of the top 50 differentially expressed genes in each cluster in HIOs ranked by highest fold change.

Biology Open • Supplementary information

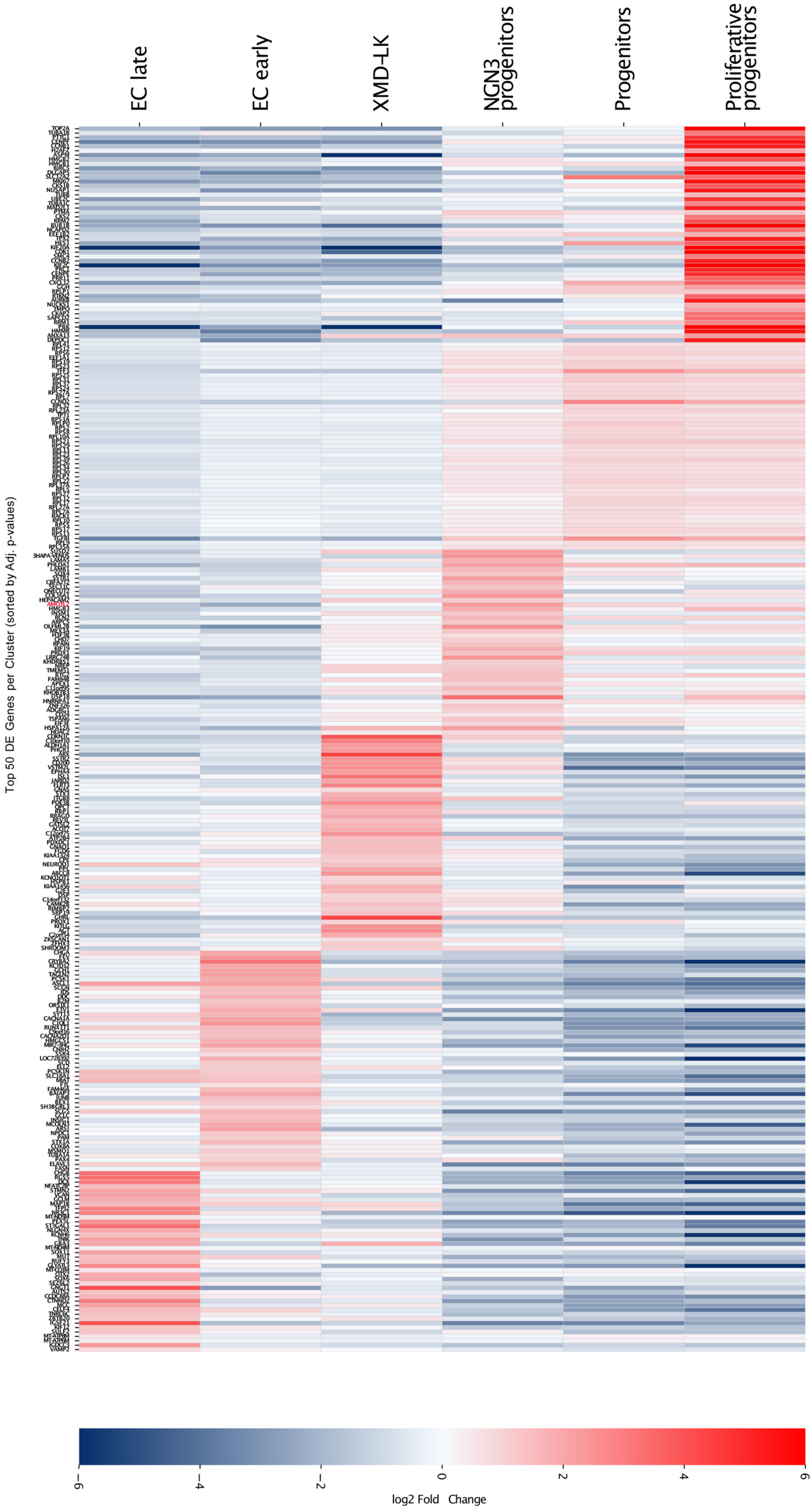

Fig. S5. **Top 50 differentially expressed genes in HIOs cell clusters (ranked by Adjusted p-values)).** Heatmap showing the fold changes of the top 50 differentially expressed genes in each cluster in HIOs ranked by lowest Adjusted p-values.

Biology Open • Supplementary information

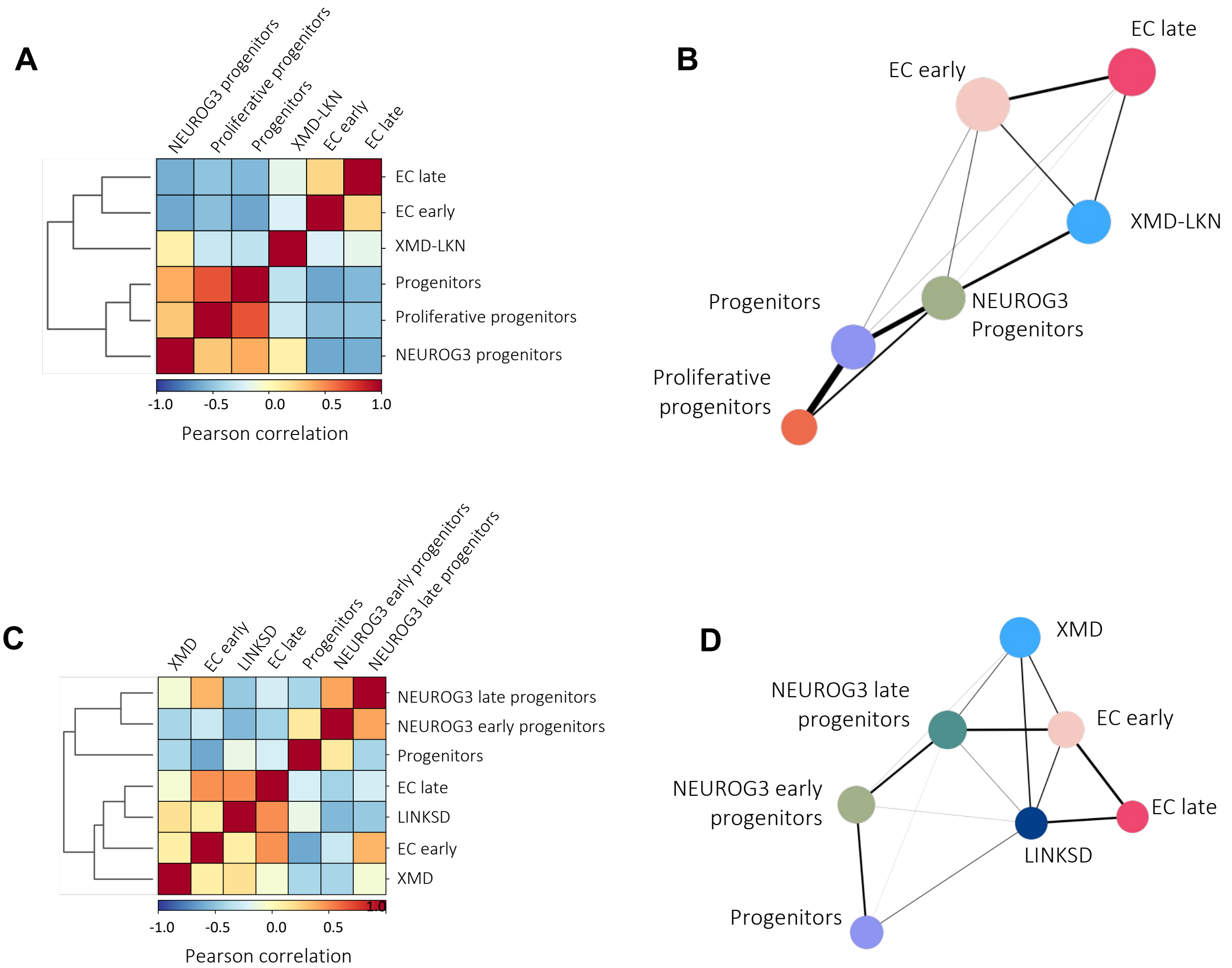

Fig. S6. Transcriptional similarities and trajectory inference identified in HIOs and tHIOs. A. Correlation map of highly variable genes between different EECs from HIOs. Color represents Pearson correlation, from blue (anti correlation) to red (perfect correlation). Progenitor clusters were more similar among them than with hormone-producing cells, from which EC cells were more similar between them than with PE cells. **B.** Relationships of EEC lineages inferred based on a measure for cluster connectivity using PAGA. Edges are weighted by significance. NGN3 P rogenitors give rise to two branches of hormone-producing cells (XMD- LK and EC). **C.** Correlation map of highly variable genes between different EECs from tHIOs. Color represents Pearson correlation, from blue (anticorrelation) to red (perfect correlation). Progenitor clusters were more similar among them than with hormone-producing cells. **D.** Relationships of EEC lineages inferred based on a measure for cluster connectivity using PAGA. Edges are weighted by significance. NGN3 late progenitors give rise to three branches of hormone-producing cells (EC, XMD, and LINKSD).

Biology Open • Supplementary information

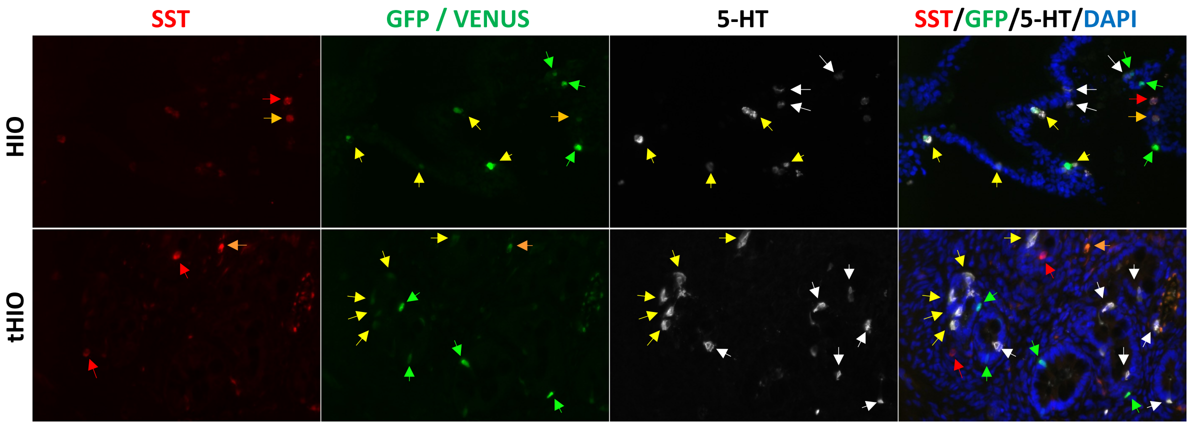

Fig. S7. Expression of EC and PE markers in tHIOs vs HIOs. Immunostaining of SST (red), GFP/Venus (green) and 5-HT (white) on HIO (upper panels) and tHIO (lower panels) cryosections. The last panels show the merge of the 3 immunostainings and nuclei stained with DAPI (blue). Yellow and orange arrows point to double positive cells for 5-HT/GFP-Venus (yellow) or SST/GFP-Venus (orange), respectively. Microscope Leica DMIRE2. Magnification: 40x.

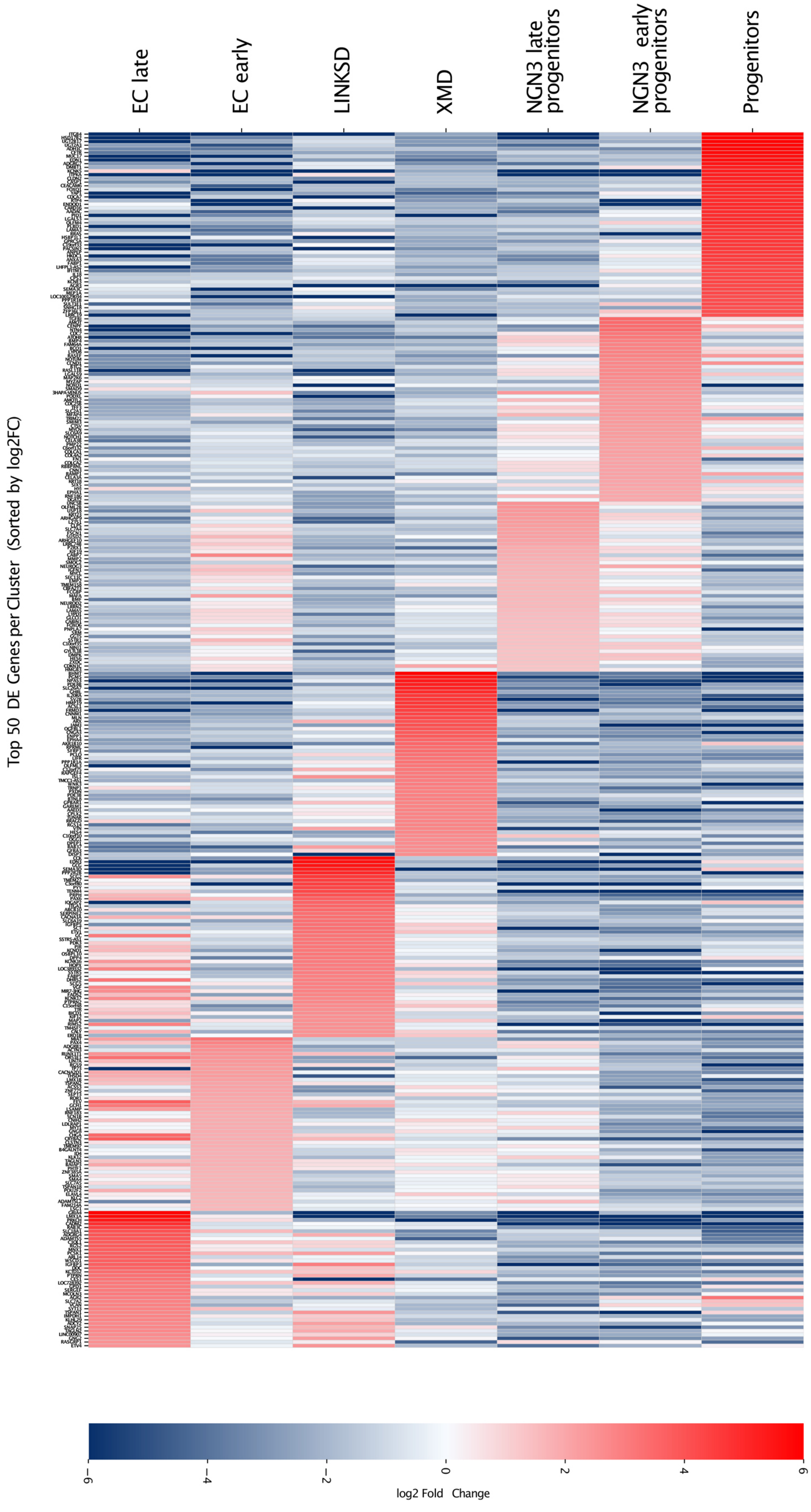

Fig. S8. **Top 50 differentially expressed genes in tHIOs cell clusters (ranked by Fold Change).** Heatmap showing the fold changes of the top 50 differentially expressed genes in each cluster in tHIOs ranked by highest fold change.

Biology Open • Supplementary information

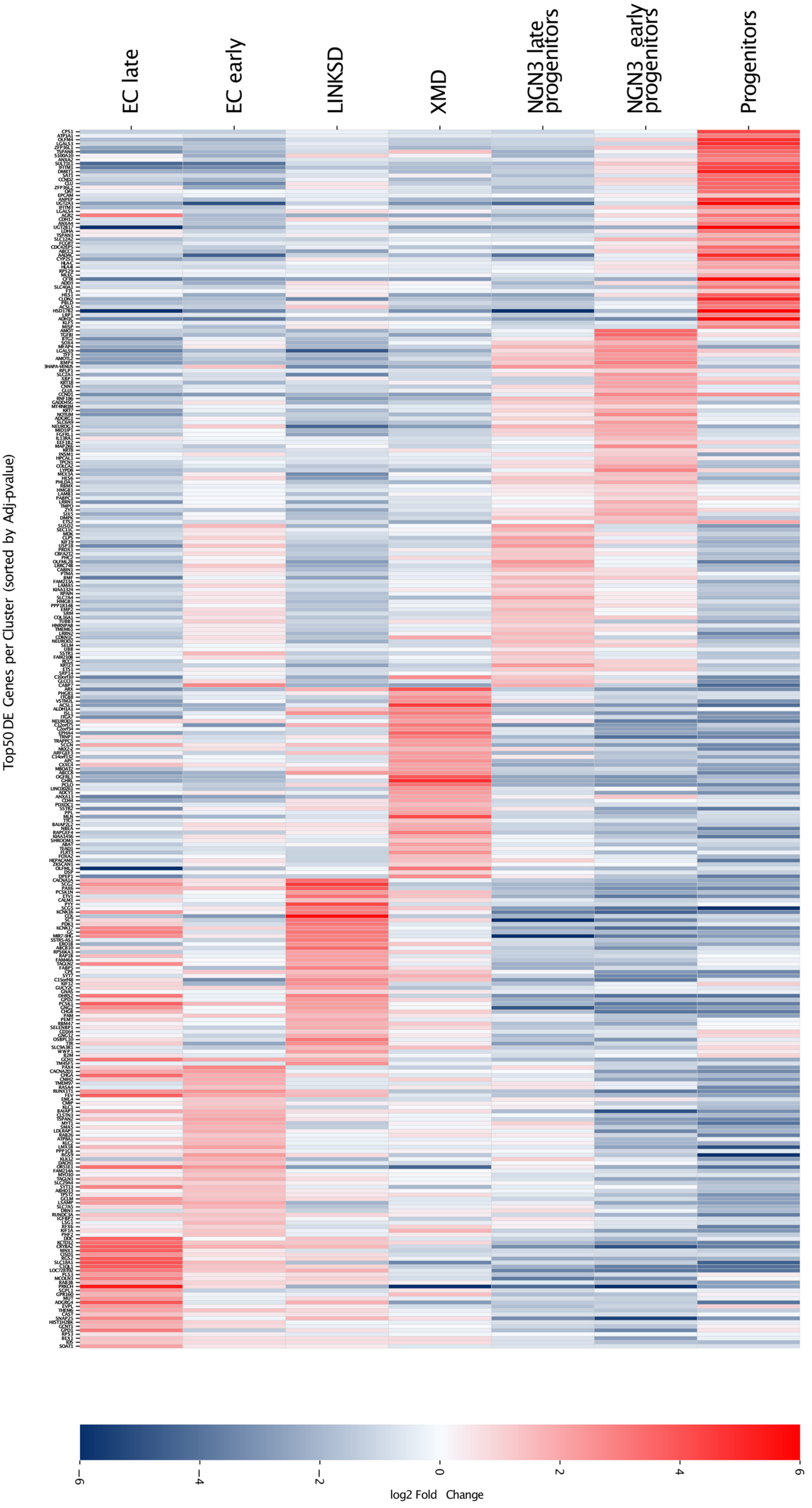

Fig. S9. **Top 50 differentially expressed genes in tHIOs cell clusters (ranked by Adjusted p-values)).** Heatmap showing the fold changes of the top 50 differentially expressed genes in each cluster in tHIOs ranked by lowest Adjusted p-values.

Biology Open • Supplementary information

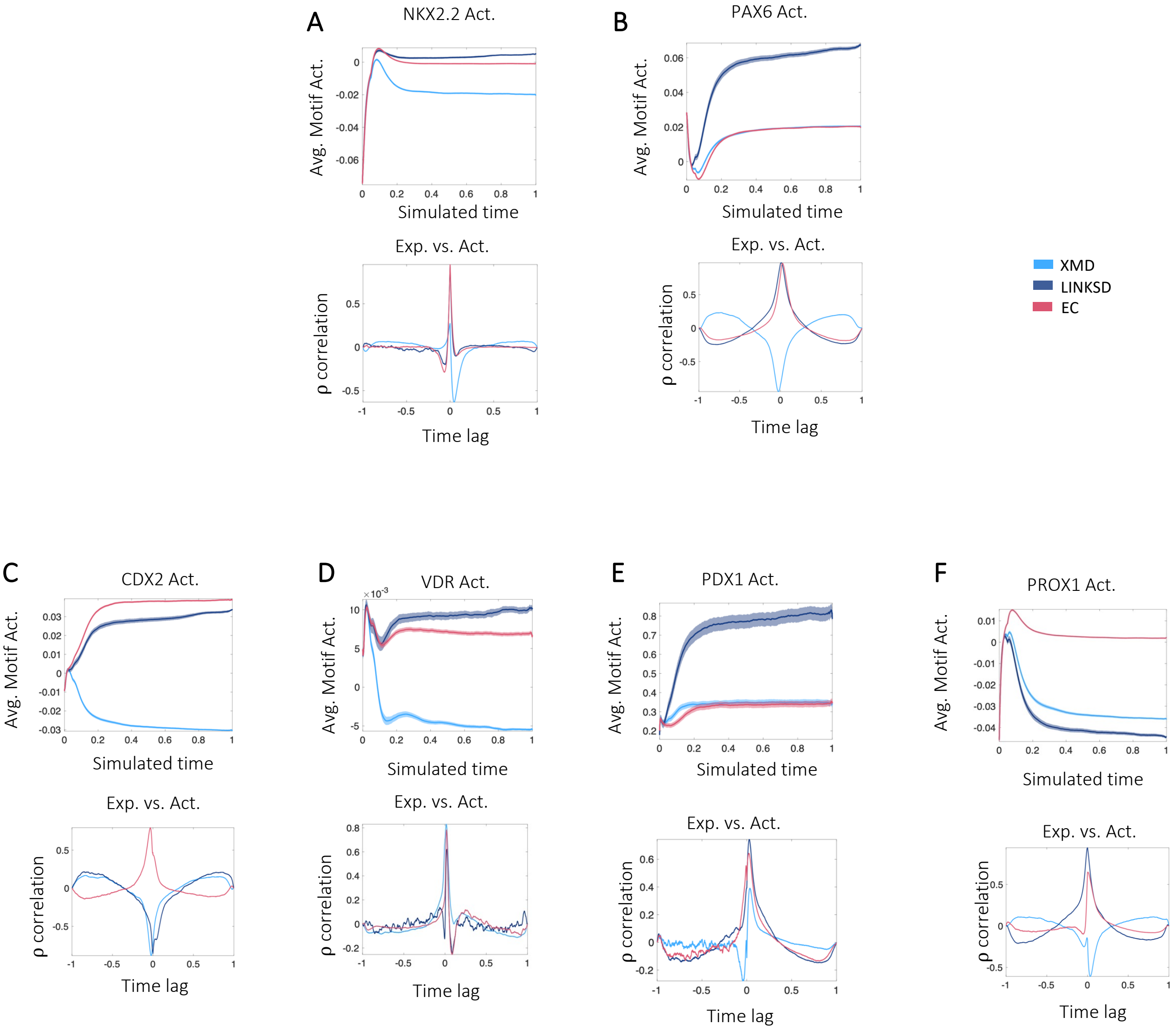

Fig. S10. Activity profile characterization in tHIOs of a selection of genes. For each transcription factor we present the average activity profile over stochastic differentiation trajectories (top panels in A-F) and the Pearson correlation between the dynamic expression and activity at different time lags (bottom panels in A-F).

Table S1. Differential motif activity analysis in tHIOs

Motif	Gene name	Ensembl ID	Model prediction
IKZF2	IKZF2	ENSG00000030419	EC
FOXJ2	FOXJ2	ENSG00000065970	EC
SPI1	SPI1	ENSG00000066336	EC
MEF2D_MEF2A	MEF2A	ENSG00000068305	EC
SREBF1_TFE3	TFE3	ENSG00000068323	EC
XBP1	XBP1	ENSG00000100219	EC
SOX6	SOX6	ENSG00000110693	EC
PLAGL1	PLAGL1	ENSG00000118495	EC
NR4A1	NR4A1	ENSG00000123358	EC
IRF2_STAT2_IRF8_IRF1	IRF1	ENSG00000125347	EC
MAX_TFEB	MAX	ENSG00000125952	EC
FOXA1	FOXA1	ENSG00000129514	EC
GRHL1	GRHL1	ENSG00000134317	EC
ETS1	ETS1	ENSG00000134954	EC
TBX3	TBX3	ENSG00000135111	EC
STAT4	STAT4	ENSG00000138378	EC
ETV6	ETV6	ENSG00000139083	EC
SMAD4	SMAD4	ENSG00000141646	EC
THRB	THRB	ENSG00000151090	EC
CXXC1	CXXC1	ENSG00000154832	EC
AIRE	AIRE	ENSG00000160224	EC
OTP_PHOX2B_LHX1_LMX1A_LHX5_HOXC4	LMX1A	ENSG00000162761	EC
EN1_ESX1_GBX1	EN1	ENSG00000163064	EC
CDX2	CDX2	ENSG00000165556	EC
SMAD3	SMAD3	ENSG00000166949	EC
NKX3-1	NKX3-1	ENSG00000167034	EC
CREB5_CREM_JUNB	JUNB	ENSG00000171223	EC
HOXB2_UNCX_HOXD3	HOXB2	ENSG00000173917	EC
RXRA_NR2F6_NR2C2	NR2C2	ENSG00000177463	EC
POU6F1	POU6F1	ENSG00000184271	EC
MAFF_MAFG	MAFF	ENSG00000185022	EC
TEAD3_TEAD1	TEAD1	ENSG00000187079	EC
HOXA4	HOXA4	ENSG00000197576	EC
SREBF2	SREBF2	ENSG00000198911	EC
STAT1_STAT3_BCL6	BCL6	ENSG00000113916	LINKSD
PROX1	PROX1	ENSG00000117707	LINKSD
ATF6	ATF6	ENSG00000118217	LINKSD
ONECUT2_ONECUT3	ONECUT2	ENSG00000119547	LINKSD
MXI1_MYC_MYCN	MXI1	ENSG00000119950	LINKSD
TWIST1_SNAI1	SNAI1	ENSG00000124216	LINKSD
GATA5	GATA5	ENSG00000130700	LINKSD
MXI1_MYC_MYCN	MYCN	ENSG00000134323	LINKSD
MXI1_MYC_MYCN	MYC	ENSG00000136997	LINKSD
POU5F1_POU2F3	POU2F3	ENSG00000137709	LINKSD

Biology Open • Supplementary information

**Motif**	**Gene name**	**Ensembl ID**	**Model prediction**
PDX1	PDX1	ENSG00000139515	LINKSD
STAT1_STAT3_BCL6	STAT3	ENSG00000168610	LINKSD
SOX11	SOX11	ENSG00000176887	LINKSD
POU5F1_POU2F3	POU5F1	ENSG00000233911	LINKSD
IRF7	IRF7	ENSG00000276561	LINKSD
PAX6	PAX6	ENSG00000007372	LINKSD_EC
SIX4	SIX4	ENSG00000100625	LINKSD_EC
SOX4	SOX4	ENSG00000124766	LINKSD_EC
NKX2-2	NKX2-2	ENSG00000125820	LINKSD_EC
GATA1_GATA4	GATA4	ENSG00000136574	LINKSD_EC
ARNT	ARNT	ENSG00000143437	LINKSD_EC
NR1I2	NR1I2	ENSG00000144852	LINKSD_EC
NR4A2	NR4A2	ENSG00000153234	LINKSD_EC
ZNF148	ZNF148	ENSG00000163848	LINKSD_EC
STAT6	STAT6	ENSG00000166888	LINKSD_EC
SMAD1	SMAD1	ENSG00000170365	LINKSD_EC
OLIG3_NEUROD2_NEUROG2	NEUROD2	ENSG00000171532	LINKSD_EC
JUN	JUN	ENSG00000177606	LINKSD_EC
HOMEZ	HOMEZ	ENSG00000215271	LINKSD_EC
CEBPA	CEBPA	ENSG00000245848	LINKSD_EC
ALX1_ARX	ARX	ENSG00000004848	XMD
PRDM1	PRDM1	ENSG00000057657	XMD
TP73	TP73	ENSG00000078900	XMD
CREB5_CREM_JUNB	CREM	ENSG00000095794	XMD
HIVEP1	HIVEP1	ENSG00000095951	XMD
DMC1	DMC1	ENSG00000100206	XMD
MYBL2	MYBL2	ENSG00000101057	XMD
DBP	DBP	ENSG00000105516	XMD
GLIS3	GLIS3	ENSG00000107249	XMD
NFKB1	NFKB1	ENSG00000109320	XMD
VDR	VDR	ENSG00000111424	XMD
TBP	TBP	ENSG00000112592	XMD
HES1	HES1	ENSG00000114315	XMD
TFCP2L1	TFCP2L1	ENSG00000115112	XMD
NR5A2	NR5A2	ENSG00000116833	XMD
NR4A3	NR4A3	ENSG00000119508	XMD
TFEC_MITF_ARNTL_BHLHE41	BHLHE41	ENSG00000123095	XMD
ATF2_ATF1_ATF3	ATF1	ENSG00000123268	XMD
SOX9	SOX9	ENSG00000125398	XMD
FOXF2_FOXJ1	FOXJ1	ENSG00000129654	XMD
NR1H2	NR1H2	ENSG00000131408	XMD
MBD2	MBD2	ENSG00000134046	XMD
TLX1_NFIC	NFIC	ENSG00000141905	XMD
TAF1	TAF1	ENSG00000147133	XMD

Biology Open • Supplementary information

Motif	Gene name	Ensembl ID	Model prediction
HMGA2	HMGA2	ENSG00000149948	XMD
POU4F1_POU4F3	POU4F1	ENSG00000152192	XMD
TCF7L1	TCF7L1	ENSG00000152284	XMD
CREB3L1_CREB3	CREB3L1	ENSG00000157613	XMD
ATF2_ATF1_ATF3	ATF3	ENSG00000162772	XMD
ZNF691	ZNF691	ENSG00000164011	XMD
PBX3	PBX3	ENSG00000167081	XMD
EMX2	EMX2	ENSG00000170370	XMD
IRX2	IRX2	ENSG00000170561	XMD
AHR_ARNT2	ARNT2	ENSG00000172379	XMD
ZBTB33_CHD2	ZBTB33	ENSG00000177485	XMD
GATA2	GATA2	ENSG00000179348	XMD
ZBTB18	ZBTB18	ENSG00000179456	XMD
ZBTB6	ZBTB6	ENSG00000186130	XMD
ZKSCAN3	ZKSCAN3	ENSG00000189298	XMD
PBX2	PBX2	ENSG00000237344	XMD
CUX1	CUX1	ENSG00000257923	XMD
KLF13	KLF13	ENSG00000275746	XMD
DLX1_HOXA3_BARX2	BARX2	ENSG00000043039	XMD_EC
FOXA3_FOXC2	FOXA3	ENSG00000170608	XMD_EC
EGR3_EGR2	EGR3	ENSG00000179388	XMD_EC
ZBTB7A_ZBTB7C	ZBTB7C	ENSG00000184828	XMD_EC
ARID5A	ARID5A	ENSG00000196843	XMD_EC
ISL1	ISL1	ENSG00000016082	XMD_LINKSD
HSF2	HSF2	ENSG00000025156	XMD_LINKSD
MECOM	MECOM	ENSG00000085276	XMD_LINKSD
RELB	RELB	ENSG00000104856	XMD_LINKSD
ARID5B	ARID5B	ENSG00000150347	XMD_LINKSD
PKNOX1_TGIF2	PKNOX1	ENSG00000160199	XMD_LINKSD
HNF4G	HNF4G	ENSG00000164749	XMD_LINKSD
ZBTB33_CHD2	CHD2	ENSG00000173575	XMD_LINKSD
ZBTB7A_ZBTB7C	ZBTB7A	ENSG00000178951	XMD_LINKSD
TEAD3_TEAD1	TEAD3	ENSG00000007866	XMD_LINKSD_EC
NR1H4	NR1H4	ENSG00000012504	XMD_LINKSD_EC
NFAT5	NFAT5	ENSG00000102908	XMD_LINKSD_EC
CDX1	CDX1	ENSG00000113722	XMD_LINKSD_EC
ARID3A	ARID3A	ENSG00000116017	XMD_LINKSD_EC
SPDEF	SPDEF	ENSG00000124664	XMD_LINKSD_EC
E2F8	E2F8	ENSG00000129173	XMD_LINKSD_EC
TCF7L2	TCF7L2	ENSG00000148737	XMD_LINKSD_EC
PRRX1_ALX4_PHOX2A	PHOX2A	ENSG00000165462	XMD_LINKSD_EC
KLF16_SP2	SP2	ENSG00000167182	XMD_LINKSD_EC
RBPJ	RBPJ	ENSG00000168214	XMD_LINKSD_EC
ZNF274	ZNF274	ENSG00000171606	XMD_LINKSD_EC
SOX13_SOX12	SOX12	ENSG00000177732	XMD_LINKSD_EC

Biology Open • Supplementary information

Table S2. Primary antibodies used in the study

Target	Host	Dilution	Reference	Manufacturer
NEUROG3	Rabbit	1/500	IGBMC	IGBMC - G. Gradwohl
Ghrelin (GHRL)	Rabbit	1/1000	IGBMC	IGBMC - C. Tomasetto
Cholecystokinin (CCK)	Goat	1/50	Sc21617	SantaCruz
GIP	Goat	1/200	Sc23554	SantaCruz
Glucagon (GCG)	Guinea pig	1/2000	A-4031-01F	Linco
Serotonin (5-HT)	Goat	1/2000	Ab66047	Abcam
Somatostatin (SST)	Rat	1/1000	MAB354	Chemicon
Secretin (SCT)	Goat	1/50	Sc26630	SantaCruz
GFP	Chicken	1/2000	ab13970	Abcam
Chromogranin A (CHGA)	Goat	1/500	sc-1488	SantaCruz
Tac1 (substance P)	Rat	1/200	MAB356	Chemicon
Peptide YY (PYY)	Rabbit	1/500	H-059-03	Phoenix
Neurotensin (NTS)	Rabbit	1/500	H-048-03	Phoenix

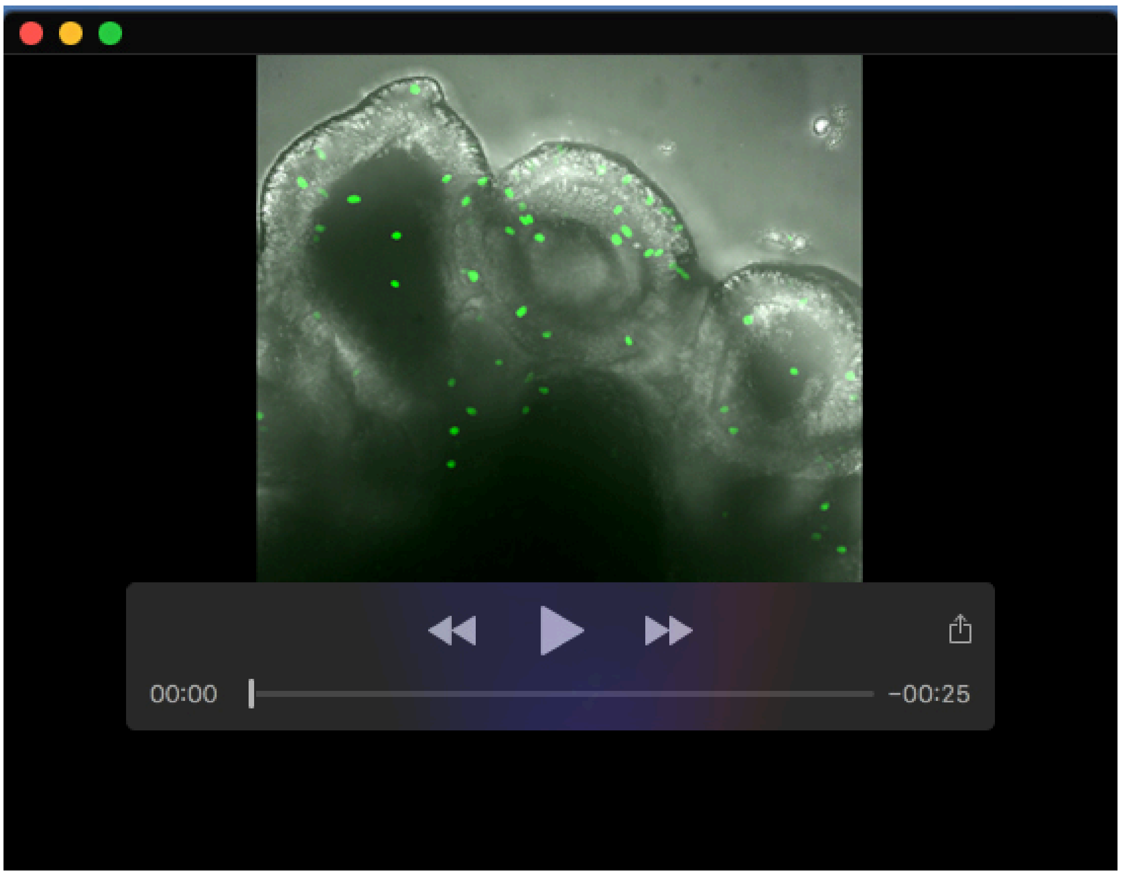

Movie 1. Time lapse video showing Venus-positive EECs in HIOs.

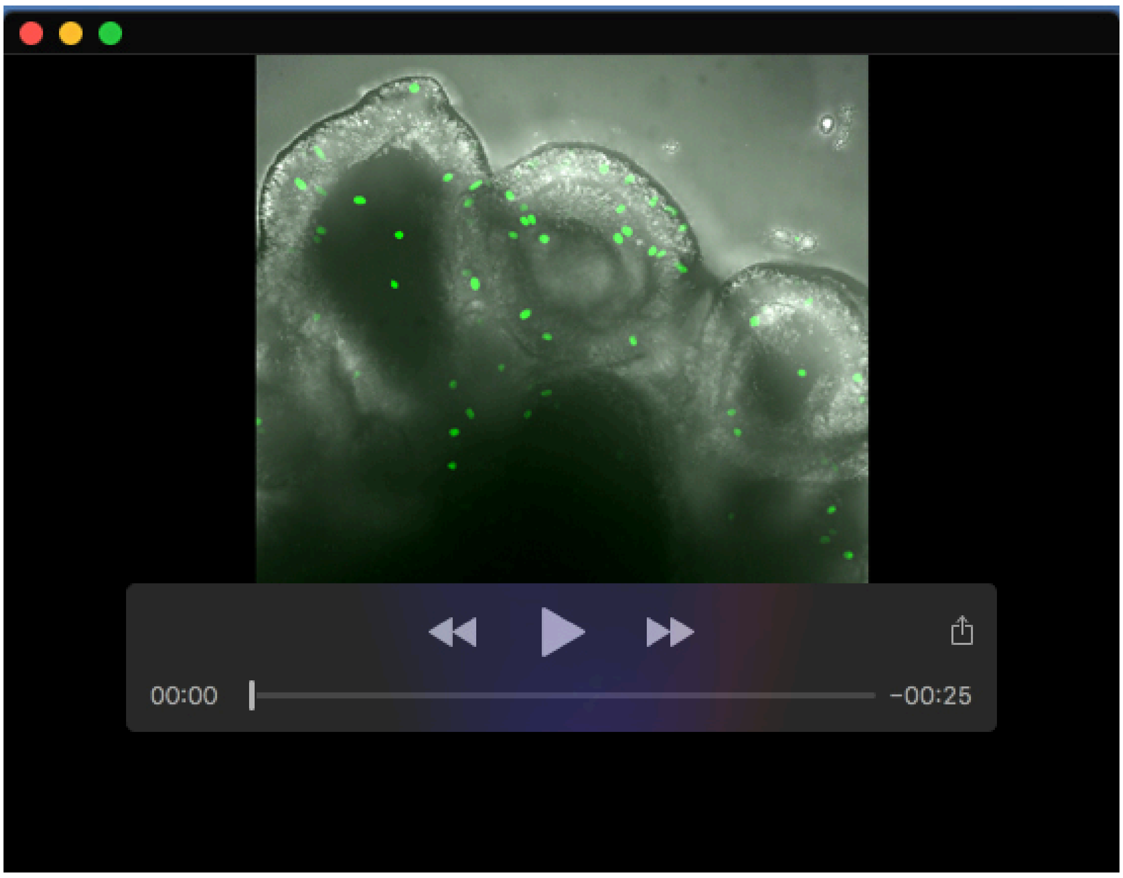

Movie 2.

Biology Open • Supplementary information
